# Galvanizing and sustaining momentum are critical to improve maternal nutrition in South Asia

**DOI:** 10.3389/fnut.2025.1498171

**Published:** 2025-01-31

**Authors:** Vani Sethi, Zivai Murira

**Affiliations:** Nutrition Section, UNICEF Regional Office for South Asia, Kathmandu, Nepal

**Keywords:** maternal nutrition, South Asia, women's nutrition, adolescent nutrition, Malnutrition, call to action

## Introduction

The South Asia region is falling significantly short of achieving the Sustainable Development Goal (SDG-2) nutrition targets by 2030 (1). While there has been some progress in the last decade in select nutrition indicators such as exclusive breastfeeding (2012: 47%, 2022: 60%) and stunting among children under five (2012: 40%, 2022: 30%), two barometers reflective of the state of women's nutrition have remain unchanged—children born with low birth weight (25%, 2012 and 2022) and women aged 15–49 years who are anemic (48%, 2012 and 2019) (1). South Asia still hosts 114 million underweight girls and women (50% of the global burden), while a rise in overweight and obesity now also affects 20% of this group in the region (2). Clearly, despite several bouts of intentional efforts by governments, multilateral organizations and civil society, progress to tackle poor maternal nutrition in South Asia has not been swift enough.

To restate the fundamentals, poor maternal nutrition is a key concern because it perpetuates multigenerational cycles of malnutrition. It is a key driver of *in-utero* malnutrition resulting in children born with low birth weight, which in turn is associated with faltered growth in infancy and future risks of developing diabetes and obesity (3). There is compelling evidence that poor maternal nutrition is caused by interrelated drivers rooted in social injustice—poverty, harmful social and gender norms, low status of women, and low women's self-efficacy are at play alongside the harsh realities of gender segregation in labor markets, wage gaps and time poverty. These experiences remain root drivers of unequal opportunities for women and girls, denying them the power and resources to access nutrition and health services and make choices about what and how much to eat—which is often last and least (4–7). To compound these difficulties, as many as 28 per cent of young women are married as children in South Asia and three in four child brides give birth while they are still adolescents, with these girls experiencing compromised agency and increased risks to birth outcomes and for their own nutrition (2, 8).

To achieve the goal of ensuring women have access to nutritious diets, nutrition services and positive nutrition practices, programmes should include a package of five essential nutrition actions: (i) access to fortified nutritious foods; (ii) micronutrient supplementation; (iii) nutrition information, education and counseling; (iv) safeguards against infections; and (v) healthy weight gain monitoring, nutrition risk screening and services for those most at-nutritional-risk at individual and population levels (cash, food vouchers, food rations and balanced energy and protein supplements). While most countries in the region do have strong policy and programme frameworks for delivering nutrition actions for pregnancy (Table 1), effective coverage of programmes remains low. About one in three women and girls in South Asia do not receive an antenatal check-up in the first trimester and in most countries in the region < 50% of women consume iron supplementation for at least 90 days of their pregnancy (9). Existing maternal service delivery platforms have programmatic challenges that constrain the availability of essential maternal nutrition services. To name but a few: (1) Priority is still accorded to reducing maternal mortality and not morbidity. Focus remains on delivering interventions to reduce child mortality and severe maternal anemia, but not on maternal morbidity or services available at women's life stages beyond pregnancy. (2) A lack of operational know-how on enacting time-efficient workflows to deliver all constituents of nutrition services at maternal and child contact points (10). (3) A cadre of trainers who understand nutrition and dietetics to support the maternal nutrition component of medical training is missing. (4) When nurses' and health providers' training takes place, the nutrition component is often weak or neglected (11). (5) Women who are thin, short, anemic, obese or with depression require “extra care” (12, 13) but this is often absent due to a lack of localized operational guidance for screening and management (10). At the planning level, there are opportunity gaps in including essential nutrition items (supplies, training, human resources, cadre, monitoring and research) in sectoral plans and budgets and missed opportunities to integrate height-weight gain monitoring, nutrition screening, macro and micronutrient supplementation, counseling and special care for nutritional risks for women into the same platforms that also reach children (14, 15). (7) Finally, despite increased attention to the need to address preconception nutrition, this is rarely provided owing to a lack of robust delivery platforms or large-scale implementation exemplars to provide the resources and programmatic know-how to cater for a large population in need.

**Table 1:** Availability of policies and programmes for delivering essential nutrition actions in pregnancy across South Asia.

Yes policy, yes program universal											
Yes policy, program in some geographies not universal											
Yes policy, no program											
No policy, no program											
Intervention not relevant to context	NA										
**Domain**	**Intervention**	**Nutrition intervention included in policy and programmes**								**# countries with policy**	**# countries with universal programme**
		**Afghanistan**	**Bangladesh**	**Bhutan**	**India**	**Maldives**	**Nepal**	**Pakistan**	**Sri Lanka**		
Nutrition information, education and counseling	Nutrition information and education on healthy eating physical activity, reduce caffeine/tobacco intake, seeking access to services, family planning									8	8
Healthy weight gain monitoring	Gestational weight gain monitoring and identification of flag signs (no, less or excessive weight gain)									8	8
Access to essential micronutrients	Iron folic acid supplementation (IFA)									8	8
	Multiple micronutrient supplements (MMS)									6	2
	Folic acid supplementation in first trimester									6	4
	Calcium supplementation (context specific)									8	6
Infection prevention (context-specific)	Deworming prophylaxis, in areas with worm infestation									6	3
	Provision of bed nets in malaria endemic areas									6	6
Nutrition status screening and interventions benefiting those at-nutritional risk at population level	Nutrition risk screening (underweight, adolescent, overweight)									0	0
	Nutrition risk specific counseling									0	0
	Social protection interventions (take home ration/ cash/ balanced energy protein supplementation)									5	3
	Anemia screening and treatment									8	8

Source: UNICEF (9).

To accelerate improvements in maternal nutrition—before, during and after pregnancy—the United Nations Children's Fund Regional Office for South Asia convened a regional conference on “Nourishing South Asia: Scaling-up equitable nutritional care for girls and women in South Asia,” from 18–20 September 2023 in Kathmandu, Nepal (16). The conference brought together 120 stakeholders from the eight countries that encompass the South Asia region (Afghanistan, Bhutan, Maldives, Bangladesh, Nepal, India, Pakistan and Sri Lanka) to take stock of countries' progress against regional commitments made in 2018 and discuss challenges and shifts needed to accelerate this progress. Details of the conference methodology have been described elsewhere (17). Briefly, the participants included senior government policy decision-makers, researchers, implementation champions, jurists, United Nations partners and development partners working on adolescent and women's nutrition (country delegations size was 6–16 per country). The conference format included 16 oral presentations, two panel discussions, a marketplace with 22 posters from the eight countries showcasing on-ground experiences and open space technology-based participatory group discussions. Each country delegations used open space methodology (18) methodology to discuss existing policies and programmes against each of the five essential nutrition actions. For those interventions which had a policy and programme, using a rubric provided (Supplementary File 1a) the country delegations identified systems bottlenecks in programme delivery, which have been described in Supplementary Files 1b–d; identified priority country actions and framework for action, which was consolidated into a call to action, which has been described below.

## 2023 regional call for five actions for improving maternal nutrition

### Develop national plans with commensurate budgetary allocations

These plans and budgets should be developed with clear targets to foster acceleration and guarantee delivery and coverage of a package of essential nutrition actions for adolescent girls and women—before, during and after pregnancy. Investments will be needed from multiple sectors, especially education, health, social protection and food systems. There is a need to increase investments to improve food environments in order to protect women from nutrient-poor and unhealthy ultra-processed foods and beverages and curb the rise in overweight and obesity. Plans should account for the differential strategies needed to reach the most marginalized communities, for example by increasing the reach of social safety nets through food assistance, cash transfers and maternity benefits which target economically vulnerable women.

### Implement solutions that are not “to” women but “through and for” women

Leveraging women's movements and coalitions will enhance the visibility of women's nutrition rights within the broader women's rights agenda. This will include accelerating multi-sector actions that address the harmful gender and social norms that underlie maternal malnutrition and especially target those that work toward keeping girls in school, delay age at marriage and strengthen family planning (to delay age at first pregnancy and reduce the number of pregnancies).

### Review and update service delivery intervention packages and toolkits to ensure comprehensiveness and alignment with global guidelines/recommendations to address all forms of malnutrition

Service delivery implementation strategies should be periodically reviewed and refined by incorporating learning from systems bottleneck analyses and systems research on “what works” at scale. Introducing innovative products with proven effectiveness such as Multiple Micronutrient Supplements (MMS) and delivering them at-scale within routine government systems offers one path to success in addressing the high burden of micronutrient deficiencies in pregnancy (19). To enhance the impact of these programmes, it would also be useful to design, develop and implement a minimum nutrition package for preconception care and women's health before, between and beyond pregnancies, delivered through maternal health, family planning and women empowerment platforms using available evidence and learnings from the region (20–22).

### Be intentional in scaling up efforts to address social and geographical inequalities that slow progress in nutrition, especially among girls and women living in the most challenging circumstances

This would entail identifying and working to close service delivery gaps, particularly at the subnational level. Extra attention and targeted nutrition action should be provided to reach malnourished adolescent girls and women who are at economic, social or geographic disadvantage. Social enterprises offer many opportunities to narrow inequities in nutrition outcomes by leveraging research and development capabilities, infrastructure, and capital from the private sector, reaching those consumer segments that could afford to buy low-cost products or services and cross-subsidizing products and services for nutrition by generating surplus from for-profit activities (23). e.g., women-led social enterprises in Afghanistan, India, Bangladesh and Nepal have worked to link “field to plate.” Setting up micro-enterprises, establishing market linkage and food fortification and processing units, community cooking, and creating grain and seed banks. These approaches aim to enhance livelihoods, agricultural practices, and household food security. They have undertake activities to establish private clinics to provide primary healthcare support to women as their right (23, 24). In humanitarian settings, creating friendly spaces for girls and women may offer the best entry point for integrated programming, particularly in contexts where movements are restricted, and girls and women are confined to their homes (25).

### Invest in knowledge—data and systems research, alliances and cross-border sharing

This would entail strengthening survey data systems and routine programme monitoring systems to close data gaps and improve the quality and timeliness of data for tracking nutritional status and the coverage of interventions. Promoting transparency in how this data is used and disseminated will further ensure that progress in implementation of strategies are both accessible and accountable to the communities they aim to serve. Strengthening academic–government collaboration in evidence-based policymaking and promoting exchange of knowledge and experience both within and between countries in South Asia can be implemented to foster support networks and a culture of cross-learning within the region to improve girls' and women's nutrition.

## A year since the 2023 call to action

Each country identified their priority action(s) during the 2023 regional conference (17). UNCEF country offices with other development partners have been supporting national/sub-national governments to mobilize the required political, technical, and financial commitments to execute priority actions identified in regional conference (Supplementary Files 1c, d) through acceleration plans/strategies. In September, 2024—a year since the conference- UNICEF released a stock-take report—*Progress and Promise: Nourishing girls and women in South Asia* (26) to capture nineteen examples of on-ground implementation since the call to action. These examples serve as testimony to the ongoing efforts across the region to improve access to essential nutrition actions for accelerating maternal nutrition. Briefly, six countries in region have initiated integration of preventive multiple micronutrient supplementation in antenatal care (Afghanistan, Bangladesh, Bhutan, Nepal and Sri Lanka). Afghanistan and Pakistan have initiated cash transfer to women leveraging poverty alleviation platforms. Indian state of Maharashtra and Srilanka have strengthened and scaled-up preconception care programmes.

On-ground implementation was further propelled through launch of global maternal nutrition acceleration plan with assured financing through child nutrition matched fund (19) for 15 countries globally, which include five countries from South Asia.

## Conclusion

South Asia remains the make-or-break region to turn around the global nutrition crises affecting girls and women and accelerate progress on the 2030 SDG nutrition targets. We urge governments in the region and their development partners to respond to the call to action and put “women at the centre” of convergent multi-sector nutrition solutions. Countries should seize the opportunity to tailor programmes to manage different nutritional risks, strengthen their focus on integrating preconception nutrition into maternal nutrition programmes, and address the gender inequalities that prevent women accessing nutritious diets, nutrition services and positive practices. Improving girls and women's nutrition is critical for accelerating progress on all SDG nutrition targets. We must not wait for another call to action to state once again what has already been asserted this decade—tackling women's malnutrition matters for nourishing South Asia!
