# Supplementary material for: Galvanizing and sustaining momentum are critical to improve maternal nutrition in South Asia
Source: Front Nutr. 2025 Jan 31;12:1498171. doi: 10.3389/fnut.2025.1498171 (PMC11825339; doi:10.3389/fnut.2025.1498171)
Supplement: Supplementary file 1 [file Table_1.docx]

**Galvanizing and sustaining momentum are critical to improve maternal nutrition in South Asia**

Vani Sethi^1†*^ and Zivai Murira^1†^

**Supplementary file**:

S1: Systems bottleneck classification criteria for each system building block

S1b: Number of countries with a programme but facing significant/moderate bottlenecks or without a programme for delivering essential nutrition actions in pregnancy across South Asia

S1c: Priority actions identified by countries based on the bottleneck analyses at the 2023 regional conference

S1d: Framework for action for improving maternal nutrition

**S1: Systems bottleneck classification criteria for each system building block**

| **System building block** | **Classification criteria for severity of bottleneck** | | | |
| --- | --- | --- | --- | --- |
|  | **Significant bottleneck** | **Moderate bottleneck** | **Mild bottleneck** | **No bottleneck** |
| 1. **Legislation and policies** |  |  |  |  |
| Are the policies/ legislations been translated into a program with operational guidelines (including financial guidelines, funded by government)?   - *Is there a legislation/ policy available to implement the intervention?* - *Has the legislation been translated into a program with operational guidelines (including financial guidelines, funded by government)?* | Only pronouncement has been made but no legislation (act) /Policy | Legislation/policy available, translated into programme with no operational framework/ guidelines and grossly inadequate financial guideline to support implementation | Legislation/policy available, translated into programme with an operational guideline in place but insufficient related to implementation | Legislation/policy available, translated into programme with an operational guideline in place with financial guidelines/costs to support implementation |
| 1. **Leadership, governance, and coordination** |  |  |  |  |
| ***If*** “yes” for Q1 then, is there leadership at national level to oversee targets and review the program with institutional architecture and administrative governance to support implementation, collaboration within and across sectors/ stakeholders/ advocates/ civil society?   - *Is there a national level/highest level leadership and coordinating mechanism which meets regularly overseas targets and comprehensive progress review of the [programme]?* - *Is there a national level/highest level leadership and coordinating mechanism which meets regularly overseas targets and comprehensive progress review of the [programme]?* - *Are there administrative governance processes established for collaboration/ consensus seeking/ stocktake for implementation of programme (within health sector, across sectors, across stakeholder groups)?* - *Is the institutional system/architecture in place to support programme implementation?* - *Are performance evaluation mechanisms in place and functional to establish accountability in the system?* - *Is there a presence of high-level advocates/civil society participation to ensure people's voices and accountability for duty bears?* | Leadership and coordinating mechanism not in place for implementation | Leadership and coordinating mechanism in place, but 2 or more challenges in implementation of established processes and systems (regarding coordination, collaboration/coalition, performance evaluation, advocacy) | Leadership and coordinating mechanism in place, but at least one challenge in implementation of established processes and systems (regarding coordination, collaboration/coalition, performance evaluation, advocacy) | Leadership and coordinating mechanism in place and this is being implemented (established processes and systems for coordination, collaboration/coalition, performance evaluation, advocacy) |
| 1. **Essential commodities and supplies** |  |  |  |  |
| Are there national standards, policies in place with respect to procurement and supply of the required drugs and equipment for the interventions? Government-owned, financed and tracking and management of stockouts?   - *Are there national standards and policies in place with respect to the required drugs and equipment?* - *Is in country procurement systems government-owned and -financed for procurement and delivery of commodities and supplies?* - *Are the procedures and mechanisms for storage and distribution of commodities and supplies for related interventions available at levels of health system?* - *Is there a system (Logistics management information system/LMIS) in place to track and manage stockouts of commodities and supplies at all system levels?* | Standard policies for drugs and equipment are not available and systems are non-functional/ need to be effectively implemented | Standard policies for drugs and equipment are in place but challenges are faced in 2 to 3 of the following areas: system for procurement, distribution and storage, systems to manage stockouts | Standard policies for drugs and equipment are in place with appropriate procurement systems but challenges existing in distribution and storage OR systems to manage stockouts | Standard policies for drugs and equipment are in place with a reliable system for procurement, distribution and storage, with equitable access at all levels, systems to manage stockouts |
| 1. **Budget and financing** |  |  |  |  |
| Are the programs/interventions budgeted? If “yes” then, allocated and disbursed efficiently; tracked; increased budgetary provisions over time?   - *Is there a budget allocation line for the programme which includes all interventions?* - *Is the allocative efficiency sufficient and includes all aspects of programme systems to implement the interventions?* - *Is there disbursement efficiency/ adequate to cover disparities?* - *Are there process instituted to support tracking allocation, disbursement and allocative efficiency and are the budgetary provisions increasing over time?* | No budget allocation line for interventions available | Budget line available but challenges exist in sufficient allocation, disbursement and its tracking | Budget line available, allocated sufficiently but challenges in efficient/ adequate disbursement and tracking | Budget line for interventions available, efficiently and sufficiently allocated, efficiently disbursed and processes instituted for tracking, with increasing trend in provisions |
| 1. **Data and information systems** |  |  |  |  |
| Is data collected on these interventions from national surveys and/or national information systems? If “yes” then, does the system include all relevant indicators and is used for program monitoring/quality improvement?   - *Are there specific goals/ targets for coverage assigned at the national level for the intervention?* - *Is data on interventions is collected from national surveys (e.g. DHS or equivalent)?* - *Does the national information system (e.g. Health Management Information System) reporting include relevant indicators pertaining to the specific intervention?* - *Is the data quality maintained through timeliness, periodicity and consistency?* - *Are systems in place for use of data generated from reporting/monitoring for programme review/ quality improvement/ decision-making?* | National Data and Information Systems are not in place/ non-functional | Specific goals/targets for coverage are assigned, however gaps existing in national survey or national information system to include relevant indicators and faces challenges in regular reporting, data quality and data use for decision making | Specific goals/targets for coverage are assigned, data collected through national survey and national information system systems on relevant indicators however some challenges exist around regular reporting, data quality OR data use for decision making | National coverage targets assigned, data collected through national surveys and national information systems with data quality and systems in place for ensuring use of data |
| 1. **Work force** |  |  |  |  |
| Are there defined responsibilities of service providers at all levels and their supervisors for implementing the interventions? Do capacity building plans exist? Adequate numbers?   - *Are there defined SOPs/ responsibilities of service providers and their supervisors at all levels for implementing the interventions?* - *Are at least 50% of staff trained at least once on implementation protocols in last on years?* - *Is the level of vacancies of trained service providers available to deliver interventions less than 25%?* - *Are there functional mechanisms in place for regular assessment and quality improvement of service provider performance?* | Guidelines and operational plans are not well defined/ do not exist at strategic level and operational level, with <25% staff trained, >50% vacancies | Guidelines and operational plans for service providers are available but face challenges in implementation along with two or more of the following: trained staff, level of vacancies, mechanism for performance assessment/ quality improvement | Guidelines and operational plans for service providers are available but face challenges in implementation along with any one of the following: trained staff, level of vacancies, mechanism for performance assessment/ quality improvement | Guidelines for service providers available, >50% staff trained, <25% vacancies of service providers and functional mechanisms for performance assessment/ quality improvement are in place |

The processes followed for bottleneck analysis include the following:

- Collated of relevant policy and program documents for key evidence-based interventions. These included strategies/plans/policies, national guidelines and standards, reports, reviews, assessments and national survey data.
- Validated the existence/ availability/ alignment of policy and program documents against each recommended intervention in consultation with UNICEF country office representatives.
- Reviewed relevant policy and program documents as well as national survey data to examine the bottlenecks in implementation of each intervention (for those interventions where programs do not exist, systems bottlenecks analysis was not undertaken).
- Conducted consultative meetings with relevant stakeholders from each country to examine the severity of bottlenecks. Stakeholders include representatives from UNICEF country offices, thematic area experts, academicians, and government representatives.
- Computed an average score based on the bottleneck grading given by each respondent for health system component against each intervention.
- Colour coded the relevant component to depict bottleneck severity, based on the average score.

S1b: Number of countries with a programme but facing significant/moderate bottlenecks or without a programme for delivering essential nutrition actions in pregnancy across South Asia

| No country | |  |  |  |  |  |  |  |  |  |
| --- | --- | --- | --- | --- | --- | --- | --- | --- | --- | --- |
| 1-2 countries | |  |  |  |  |  |  |  |  |  |
| 3-4 countries | |  |  |  |  |  |  |  |  |  |
| 5-8 countries | |  |  |  |  |  |  |  |  |  |
| **Domain** | **Intervention** | | | **Number of countries facing a moderate/significant bottleneck for the systems block** | | | | | | |
|  |  |  |  | Legislation and policies | Leadership, governance, and coordination | Essential commodities and supplies | Budget and financing | Data and information systems | Work force |  |
| Nutrition information, education & counselling | Nutrition information and education on healthy eating physical activity, reduce caffeine/tobacco intake, seeking access to services, family planning | | | 1 | 1 | 3 | 4 | 8 | 4 |  |
| Healthy weight gain monitoring | Gestational weight gain monitoring and identification of flag signs (no, less or excessive weight gain) | | | 1 | 1 | 4 | 2 | 3 | 2 |  |
| Access to essential micronutrients | Iron folic acid supplementation (IFA) | | | 0 | 2 | 1 | 2 | 2 | 2 |  |
|  | Multiple micronutrient supplements (MMS) | | | 3 | 5 | 6 | 8 | 7 | 4 |  |
|  | Folic acid supplementation in first trimester | | | 4 | 5 | 4 | 5 | 7 | 5 |  |
|  | Calcium supplementation (context specific) | | | 1 | 3 | 3 | 4 | 6 | 3 |  |
| Infection prevention  (context-specific) | Deworming prophylaxis, in areas with worm infestation | | | 2 | 4 | 4 | 4 | 5 | 5 |  |
|  | Provision of bed nets in malaria endemic areas | | | 2 | 3 | 4 | 6 | 4 | 3 |  |
| Nutrition status screening and interventions benefiting those at-nutritional risk at population level | Nutrition risk screening (underweight, adolescent, overweight, anemic) | | | 0 | 1 | 2 | 1 | 3 | 1 |  |
|  | Nutrition risk specific counseling | | | 1 | 2 | 4 | 4 | 5 | 4 |  |
|  | Social protection interventions (take home ration/ cash/ balanced energy protein supplementation) | | | 3 | 3 | 5 | 6 | 5 | 5 |  |
|  | Anemia screening and treatment | | | 1 | 1 | 4 | 2 | 2 | 2 |  |

**S1c: Priority actions identified by countries based on the bottleneck analyses at the 2023 regional conference**

| Countries | ADOLESCENT GIRLS | Preconception | Pregnant women | Women in postnatal |
| --- | --- | --- | --- | --- |
| **Afghanistan** | Work with social and behavioural change section to develop materials to increase weekly iron and folic acid supplementation compliance and demand generation. | Advocate within the nutrition departments to initiate work on preconception (with the support of UNICEF ROSA). | Scale up of multiple micronutrient supplementation. | Work with health departments to strengthen community health structures and increase access to postnatal care services. |
| **Bangladesh** | Address data gaps (survey data, programme data and surveillance system).  Universalize nutrition service coverage through in-school and out-of-school platforms, such as community clubs. | Integrate nutrition in existing family planning systems for newly married couples. | Track 70% of pregnant women for gestational weight gain monitoring and promotion of antenatal care visits, including diet and multiple micronutrient supplementation in both rural and urban areas. | Integrate postnatal care for mothers and neonates in the newborn package.  Ensure effective coverage of the community-based engagement package. |
| **Bhutan** | Address the triple burden of malnutrition and promote healthy dietary practices through nutrition education. | Develop and implement a minimum nutrition package for preconception care. | Launch multiple micronutrient supplementation and gestational age-wise counselling in antenatal care. | Ensure postnatal multiple micronutrient supplementation and nutrition counselling for continuation of maternal nutrition care. |
| **India** | Review and strengthen multisectoral platforms to provide information and services to adolescents, anchored by the Ministry of Health. | Make an investment case for policymakers (bottom-up) – Engage policymakers to review preconception studies and programmes at subnational level to take those learnings to scale. | Screen, identify and manage at-risk pregnant women. | Refine the postnatal package to include psychosocial support and screening and managing at-risk mothers. |
| **Maldives** | Advocate and introduce multiple micronutrient supplementation via school platforms, health facilities, non-governmental organizations and youth groups. | Design and roll out a minimum package through context-responsive platforms. | Strengthen delivery of the antenatal care intervention package to include a comprehensive set of nutrition services. | Strengthen postnatal care to include a comprehensive set of nutrition services with home visits. |
| **Nepal** | Strengthen adolescent-friendly nutrition services within community and school platforms. | Define the target group and integrate preconception nutrition intervention within family planning platforms. | Deliver a comprehensive nutrition package, including screening and management of nutrition risks. | Strengthen postnatal care to include a comprehensive set of nutrition services linked to maternal registration. |
| **Pakistan** | Keep girls in school and ensure access to a minimum package of health and nutrition services (5 strategies X 5 interventions framework). | Advocate for formalizing preconception counselling within marriage registration and sensitization to health and nutrition. | Link conditional cash transfers to antenatal care visits, iron and folic acid/multiple micronutrient supplementation compliance and tetanus toxoid vaccination. | Link conditional cash transfers to institutional delivery, postpartum psychosocial and family planning support and counselling on maternal, infant, and young child feeding practices. |
| **Sri Lanka** | Enable adolescents to initiate interventions to promote their own nutrition). | Identify a strategy to deliver preconception care packages to all married and unmarried women/couples. | Strengthen the health system to enhance health-seeking behaviour to address maternal morbidities to reduce low birthweight and preterm deliveries. | Carry out postpartum weight retention monitoring and interventions via existing systems. |

Source: UNICEF 2024a

**S1d2:** Regional framework for action for delivering an essential maternal nutrition intervention package for women.

**GOAL**

**OBJECTIVE**

**INTERVENTIONS**

**STRATEGIES**

1**. Science-driven advocacy** for strengthened policies, financing and accountability for prevention of malnutrition and anaemia in pregnant women.

**2. Strengthening guidelines with costed plans and institutional architecture** to improve delivery of maternal nutrition services through food, health, and social protection systems.

**To protect and promote diets, services and positive nutrition practices that support optimal nutrition for women during pregnancy and breastfeeding**

**Country has policies, strategies and programmes that support optimal maternal nutrition**

**Country adopts services and practices that support optimal maternal nutrition**

**Nutrition information, education & counselling**

Gestational-month specific counselling on healthy eating, physical activity, reducing caffeine/tobacco intake, and seeking access to services.

Gestational weight gain monitoring and identification of flag signs (no, less or excessive weight gain).

Multiple micronutrient supplementation.

Iron and folic acid supplementation.

Deworming prophylaxis, in areas with worm infestation.

Nutrition risk screening (underweight, adolescent, anaemic).

Nutrition risk specific counselling

3. **Increase the capacity and support to service providers** at facility and community levels to deliver maternal nutrition services through community partnerships.

**4. Increase supplies of essential commodities** to meet growing demand and strengthen systems to prevent stockouts.

**5. Harness use of data and generate evidence** (implementation research) to inform policy and programme decisions, and strengthen accountability.

**Screening and management of nutrition**

**Infection prevention**

**Access to essential micronutrients**

**Healthy weight gain monitoring**
